# 维奈克拉联合去甲基化药物治疗较高危骨髓增生异常综合征83例疗效与安全性分析

**DOI:** 10.3760/cma.j.cn121090-20231207-00296

**Published:** 2024-03

**Authors:** 柳 刘, 凤 和, 衍 徐, 涛 李, 亚飞 李, 平 汤, 玲 孙

**Affiliations:** 郑州大学第一附属医院血液科，郑州 450052 Department of Hematology, The First Affiliated Hospital of Zhengzhou University, Zhengzhou 450052, China

**Keywords:** 骨髓增生异常综合征, 维奈克拉, 疗效, 影响因素, Myelodysplastic syndromes, Venetoclax, Efficacy, Influencing factor

## Abstract

**目的:**

评估维奈克拉（VEN）联合去甲基化药物（HMA）治疗较高危骨髓增生异常综合征（HR-MDS）的疗效和安全性，并分析可能影响疗效的因素。

**方法:**

回顾性分析2019年11月至2023年5月在郑州大学第一附属医院血液科确诊的83例HR-MDS患者的临床资料，所有患者在治疗期间均应用过VEN+ HMA治疗。生存曲线采用Kaplan-Meier法绘制，组间生存比较采用Log-rank检验。

**结果:**

83例HR-MDS患者中，男性51例（61.4％），中位年龄57（15～82）岁。其中，初始治疗MDS患者45例（54.2％），应用HMA≤4个疗程患者23例（27.7％），HMA治疗失败15例（18.1％）。中位随访10.3（0.6～34.4）个月，总体反应率（ORR）62.7％（52/83），其中18例（21.7％）获完全缓解（CR），14例（16.9％）获骨髓完全缓解（mCR）并血液学改善，20例（24.1％）获mCR。初始治疗、应用HMA≤4个疗程、HMA治疗失败3组患者的ORR分别为66.7％、60.9％、53.3％（*P*＝0.641）。中位总生存（OS）期14.6（95％*CI* 7.2～22.0）个月，中位无进展生存（PFS）期8.9（95％*CI* 6.7～11.1）个月。多因素分析显示，碱性磷酸酶（ALP）≥90 U/L（*OR*＝14.574，95％*CI* 3.036～69.951，*P*＝0.001）、TP53突变（*OR*＝13.052，95％*CI* 1.982～85.932，*P*＝0.008）和U2AF1突变（*OR*＝7.720，95％*CI* 1.540～38.698，*P*＝0.013）是VEN+HMA治疗无效的独立危险因素。所有患者均发生了血液学不良事件（AE），因治疗所致3～4级白细胞减少的发生率最高，为48.2％（40/83）。最常见的非血液AE为肺部感染（31.3％）。

**结论:**

VEN+HMA在初始治疗及HMA治疗失败的HR-MDS患者中均有较高的治疗反应率，ALP≥90 U/L、TP53突变和U2AF1突变是无治疗反应的独立危险因素。

## Abstract (en)

**Objective:**

This study aimed to evaluate the efficacy and safety of venetoclax（VEN）combined with hypomethylating agents（HMA）in the treatment of higher-risk myelodysplastic syndromes（HR-MDS）and analyze the factors influencing their therapeutic effect.

**Methods:**

The clinical data of 83 patients with HR-MDS who were diagnosed at the First Affiliated Hospital of Zhengzhou University between November 2019 and May 2023 were retrospectively analyzed. All patients were treated with VEN combined with HMA. The Kaplan-Meier method was used to depict the survival curves, and the log-rank test was used to compare survival between the groups.

**Results:**

The median age was 57（15–82）years old, and 51 patients（61.4%）were male. Forty-five patients（54.2%）were initially treated with HMA, 23（27.7%）received ≤4 cycles of HMA, and 15（18.1%）demonstrated HMA failure. At the median follow-up of 10.3（0.6–34.4）months, the overall response rate（ORR）was 62.7%（52/83）, including 18 patients（21.7%）with a complete response（CR）, 14（16.9%）with a bone marrow CR（mCR）with hematological improvement, and 20（24.1%）with a mCR. The ORR of patients with initial treatment, ≤4 HMA cycles, and HMA failure were 66.7%, 60.9%, and 53.3%, respectively（*P*＝0.641）. The median overall survival time was 14.6（95% *CI* 7.2–22.0）months, and the median progression-free survival time was 8.9（95% *CI* 6.7–11.1）months. The multivariate analysis showed that serum alkaline phosphatase（ALP）≥90 U/L（OR＝14.574, 95% *CI* 3.036–69.951, *P*＝0.001）, TP53 mutation（*OR*＝13.052, 95% *CI* 1.982–85.932, *P*＝0.008）, and U2AF1 mutation（*OR*＝7.720, 95% *CI* 1.540–38.698, *P*＝0.013）were independent risk factors for poor efficacy of VEN combined with HMA. Hematological toxicity occurred in all patients, and the incidence of treatment-induced grade 3–4 leukopenia was 48.2%（40/83）. Infection was the most common non-hematological adverse event, mainly pulmonary infection（31.3%）.

**Conclusion:**

VEN combined with HMA had a high response rate in patients with HR-MDS, both at initial treatment and with HMA failure. ALP ≥ 90 U/L, TP53 mutation, and U2AF1 mutation were independent risk factors for non-response to treatment.

骨髓增生异常综合征（MDS）是一组异质性髓系肿瘤性疾病，其基本特征是骨髓造血细胞发育异常、外周血一系或多系血细胞减少以及高风险向急性髓系白血病（AML）转化[1]–[3]。较高危MDS（HR-MDS）目前缺乏有效的治疗手段，患者中位生存期仅1.5年[1]。包括地西他滨（DEC）和阿扎胞苷（AZA）的去甲基化药物（HMA）虽然可使部分患者生存获益，但总体治疗反应率低，且多数患者终将面临复发。HMA治疗失败后的患者预后极差，只有不到三分之一的患者可存活1年以上[4]–[7]。异基因造血干细胞移植（allo-HSCT）是唯一有可能根治MDS的方法，但由于患者高龄、合并症等因素，仅少数患者可接受allo-HSCT[2],[8]–[9]。

维奈克拉（VEN）是一种口服的B细胞淋巴瘤-2（Bcl-2）抑制剂，与HMA联合治疗MDS，总体治疗反应率较高，并可延长生存期，但以VEN为基础的治疗方案的原发性和获得性耐药仍然是一个主要问题[10]–[12]。本研究中，我们回顾性评估我中心应用VEN联合HMA治疗HR-MDS的疗效和安全性，并初步探索影响疗效的相关因素。

## 病例与方法

一、病例来源

收集自2019年11月至2023年5月就诊于郑州大学第一附属医院血液科的HR-MDS且在治疗期间曾应用VEN+HMA治疗患者的临床资料，所有患者均根据《骨髓增生异常综合征中国诊断与治疗指南（2019年版）》诊断标准进行诊断[8]。纳入标准：①既往HMA治疗失败的HR-MDS患者（HMA治疗失败指经过4个疗程治疗仍无效或2个疗程治疗后疾病进展或治疗有效后疾病复发）；②应用HMA低于4个疗程，后调整为VEN+HMA治疗；③诊断明确时即开始应用VEN+HMA治疗；④所有患者在转化为AML前曾接受至少1个周期的VEN+HMA治疗；⑤所有患者的年龄均≥14岁。同时分析本中心2016年9月至2019年9月应用HMA单药治疗的HR-MDS患者的临床资料作为对照。本研究已经过郑州大学第一附属医院伦理委员会批准（批件号：2023-KY-1347-001）。

二、基因测序分析

采用二代测序的方法，检测了包括SF3B1、TET2、ASXL1、SRSF2、DNMT3A、RUNX1、U2AF1、TP53、EZH2、ZRSR2等在内的常见突变基因。变异等位基因频率最小阈值>1％。检测方法：①提取送检样本中的基因组DNA，分析目标基因蛋白质编码区域的点突变、插入或缺失突变。②采用扩增法和捕获法建库，采用NGS基因Panel检测文库构建试剂盒进行标准文库构建，在Nextseq 550上进行PE150测序，平均测序深度为2 000×。测序后的原始数据利用 NCBI、Clinvar、dbSNP（v138）、COSMIC、人基因组数据库（HG19）等进行分析，使用igv软件进行突变分析。选取Ⅰ类突变结果进行后续数据分析。

三、染色体核型分析

根据《人类细胞遗传学国际命名体制（ISCN2013）》描述核型异常，按照修订的国际预后积分系统（IPSS-R）染色体核型分组标准进行染色体核型预后分组。

四、治疗方案

VEN+HMA的具体治疗方案分为两种：①VEN+AZA：VEN 400 mg/d，d 1～14，AZA 75 mg·m^−2^·d^−1^，d 1～7；②VEN+DEC：VEN 400 mg/d，d 1～14，DEC 15～20 mg·m^−2^·d^−1^，d 1～5。当合用伏立康唑时，调整VEN剂量为100 mg/d。HMA单药治疗方案为：①AZA 75 mg·m^−2^·d^−1^，d 1～7；②DEC 15～20 mg·m^−2^·d^−1^，d 1～5。

五、疗效评估

参照《骨髓增生异常综合征中国诊断与治疗指南（2019年版）》，分为完全缓解（CR）、部分缓解（PR）、骨髓完全缓解（mCR）、血液学改善（HI）、疾病稳定（SD）、治疗失败。疗效达CR、mCR和PR的患者视为治疗有效，总体反应率（ORR）定义为：CR+mCR+PR之和。

六、安全性评价

根据《常见不良反应事件评价标准（CTCAE）》5.0版[13]，对患者每个周期用药后发生的不良事件（AE）进行分级。

七、随访

采用门诊、住院或电话联系的方式进行随访，随访截止时间为2023年6月10日。既往HMA单药治疗的患者随访截止时间为2020年2月1日。总生存（OS）时间定义为自应用维奈克拉之日起至任何原因死亡或随访截止的时间。无进展生存（PFS）定义为自应用VEN之日起至疾病进展、复发、死亡或随访截止的时间。

八、统计学处理

采用SPSS 26.0软件进行数据分析。计数资料采用例数（构成比）表示，组间比较采用卡方检验或Fisher确切概率法。疗效影响因素分析采用单因素Logistic回归分析。采用Kaplan-Meier法绘制生存曲线，组间比较采用Log-rank检验。双侧*P*<0.05为差异有统计学意义。

## 结果

一、临床特征和疗效评估

83例HR-MDS患者中，男51例（61.4％），女32例（38.6％），中位年龄57（15～82）岁。初始治疗MDS患者45例（54.2％），应用HMA≤4个疗程患者23例（27.7％），HMA治疗失败15例（18.1％）。其中，MDS伴原始细胞增多1型34例（41.0％），MDS伴原始细胞增多2型49例（59.0％）。所有患者均具有可供分析的染色体核型结果，IPSS-R评分均≥4分。根据IPSS-R分组，中危组12例（14.5％），高危组 31例（37.3％），极高危组40例（48.2％）。69例（83.1％）患者有基因测序结果，常见的突变基因（突变检出率>10％）包括ASXL1（33.3％）、U2AF1（21.7％）、NRAS（17.4％）、TET2（14.5％）、RUNX1（14.5％）、TP53（13.0％）、DNMT3A（10.1％）。

71例（85.5％）患者接受VEN+AZA治疗，中位治疗2（1～10）个周期；12例（14.5％）接受VEN+DEC治疗，中位治疗3（1～17）个周期。ORR为62.7％（52/83），其中18例（21.7％）达CR，14例（16.9％）mCR并HI，20例（24.1％）获mCR。获得治疗反应的中位时间为1.3（95％*CI* 1.0～3.3）个月。初始治疗、应用HMA≤4个疗程、HMA治疗失败3组患者的ORR分别为66.7％、60.9％、53.3％（*P*＝0.641）。对比本中心既往应用HMA单药治疗的53例HR-MDS患者，ORR为28.3％（CR 8例、mCR 5例、PR 2例），VEN+HMA的疗效明显优于HMA单药（*χ*^2^＝15.268，*P*<0.001）。

根据性别、年龄、常用的血常规和生化指标、骨髓涂片原始细胞数以及染色体核型、突变基因、预后分层、前期治疗史进行分组，单因素分析结果显示，碱性磷酸酶（ALP）≥90 U/L、TP53突变是影响VEN+HMA疗效的危险因素（表1）。

**表1:** HR-MDS患者影响VEN+HMA疗效的单因素分析

影响因素	*OR*（95%*CI*）	*P*值
女性	1.254（0.505~3.115）	0.625
年龄≥60岁	0.961（0.393~2.347）	0.930
ANC≥1.0×10^9^/L	1.311（0.537~3.201）	0.552
HGB≥60 g/L	0.553（0.146~2.089）	0.383
PLT≥50×10^9^/L	0.604（0.233~1.566）	0.300
RDW>14.5%	0.656（0.236~1.822）	0.418
MCV>100 fl	0.632（0.256~1.560）	0.319
MCH>34 pg	0.334（0.110~1.015）	0.053
铁蛋白>400 µg/L	1.665（0.599~4.628）	0.328
LDH>245 U/L	1.318（0.535~3.245）	0.549
β_2_-MG>3 mg/L	1.058（0.313~3.575）	0.928
维生素B_12_>1000 ng/L	0.972（0.337~2.808）	0.958
ALP≥90 U/L	3.536（1.196~10.455）	0.022
骨髓原始细胞比例>10%	0.558（0.216~1.444）	0.229
VEN+DEC	0.815（0.224~2.967）	0.756
IPSS-R细胞遗传学风险（极差）	1.808（0.479~6.828）	0.383
IPSS-R分层（极高危）	1.244（0.511~3.033）	0.630
TP53突变	4.677（1.050~20.745）	0.043
ASXL1突变	1.758（0.624~4.956）	0.286
RUNX1突变	2.105（0.543~8.158）	0.281
U2AF1突变	2.714（0.842~8.750）	0.095
TET2突变	0.776（0.181~3.318）	0.732
NRAS突变	0.571（0.139~2.348）	0.438
DNMT3A突变	0.727（0.130~4.063）	0.717

注 HR-MDS：较高危骨髓增生异常综合征；VEN：维奈克拉；HMA：去甲基化药物；RDW：红细胞分布宽度；MCV：平均红细胞体积；MCH：平均红细胞血红蛋白含量；β_2_-MG：β_2_微球蛋白；ALP：碱性磷酸酶；DEC：地西他滨；IPSS-R：修订的国际预后积分系统

将单因素分析中*P*<0.1的因素纳入多因素Logistic回归模型，结果显示ALP≥90 U/L、TP53突变和U2AF1突变是VEN+HMA治疗无效的独立影响因素（表2）。

**表2:** HR-MDS患者影响VEN+HMA总体反应的多因素分析

影响因素	*OR*（95％*CI*）	*P*值
MCH>34 pg	0.528（0.112~2.484）	0.419
ALP≥90 U/L	14.574（3.036~69.951）	0.001
TP53突变	13.052（1.982~85.932）	0.008
U2AF1突变	7.720（1.540~38.698）	0.013

注 HR-MDS：较高危骨髓增生异常综合征；VEN：维奈克拉；HMA：去甲基化药物；MCH：平均红细胞血红蛋白含量；ALP：碱性磷酸酶

二、安全性评估

血液学AE发生率为100.0％（83/83）, 3～4级白细胞减少、血小板减少和贫血的发生率分别为48.2％（40/83）、25.3％（21/83）和27.7％（23/83）。

最常见的非血液学AE是感染，包括肺部感染（31.3％）和肛周感染（2.4％），均为≥3级AE。其次为胃肠道反应，包括恶心和呕吐（18.1％）、便秘（16.9％）、腹泻（2.4％）。接受VEN+DEC与VEN+AZA两组患者间各类血液学AE与非血液学AE发生率差异无统计学意义（表3）。因严重血细胞减少或感染导致第2个疗程推迟（>7 d）的患者占63.8％（51/80），其中VEN+DEC组6例（54.5％，6/11），VEN+AZA组45例（65.2％，45/69），两组间差异无统计学意义（*χ*^2^＝0.120，*P*＝0.729）。因严重血细胞减少致VEN和（或）HMA用药天数缩短的患者占22.6％（12/53），其中VEN+DEC组3例（25.0％，3/12），VEN+AZA组9例（22.0％，9/41），两组间差异无统计学意义（Fisher，*P*＝1.000）。

**表3:** 第1个疗程VEN+HMA治疗HR-MDS出现的不良事件

不良反应	任何级别AE	≥3级AE			
		总体（83例）	VEN+DEC组（12例）	VEN+AZA组（71例）	*P*值
血液学AE					
白细胞减少	43（51.8）	40（48.2）	3（25.0）	37（52.1）	0.082
血小板减少	21（25.3）	21（25.3）	5（41.7）	16（22.5）	0.293
中性粒细胞减少	39（47.0）	34（41.0）	2（16.7）	32（45.1）	0.125
贫血	24（28.9）	23（27.7）	4（33.3）	19（26.8）	0.903
中性粒细胞减少伴发热	28（33.7）	28（33.7）	6（50.0）	22（40.0）	0.338
非血液学AE					
肺部感染	26（31.3）	26（31.3）	6（50.0）	20（28.2）	0.241
恶心、呕吐	15（18.1）	11（13.3）	0	11（15.5）	0.316
便秘	14（16.9）	0	0	0	-
疲乏	12（14.5）	12（14.5）	1（8.3）	11（15.5）	0.835
咳嗽	10（12.0）	0	0	0	-
腹胀/腹痛	7（8.4）	6（7.2）	1（8.3）	5（7.0）	1.000
胸闷/胸痛	5（6.0）	0	0	0	-
头晕/头痛	4（4.8）	0	0	0	-
肛周感染	2（2.4）	2（2.4）	0	2（2.8）	1.000
腹泻	2（2.4）	1（1.2）	0	1（1.4）	1.000
皮肤瘙痒	1（1.2）	0	0	0	-
耳鸣/耳聋	1（1.2）	0	0	0	-

注 VEN：维奈克拉；HMA：去甲基化药物；HR-MDS：较高危骨髓增生异常综合征；AE：不良事件；DEC：地西他滨；AZA：阿扎胞苷；-：不适用

三、进展和生存

中位随访10.3（0.6～34.4）个月，随访结束时12例（14.5％）患者进展为AML，27例（32.5％）患者死亡（其中7例死于感染性休克，5例死于脑出血，4例死于呼吸衰竭，1例死于肺出血，1例死于多脏器衰竭，余9例死因不详）。83例患者的中位OS期为14.6（95％*CI* 7.2～22.0）个月，中位PFS期为8.9（95％*CI* 6.7～11.1）个月。与本中心既往单独应用HMA治疗的53例HR-MDS相比，OS期有所延长，但差异无统计学意义（14.6个月对9.9个月，*χ*^2^＝1.176，*P*＝0.278）。

VEN+HMA治疗有效组患者的中位OS期未达到，无效组患者的中位OS期为8.9（95％*CI* 5.3～12.5）个月，差异有统计学意义（*χ*^2^＝9.982，*P*＝0.002）。VEN+HMA治疗有效组的中位PFS期为11.1（95％*CI* 7.1～15.1）个月，无效组的中位PFS期为4.7（95％*CI* 1.9～7.4）个月，差异有统计学意义（*χ*^2^＝16.527，*P*<0.001）（图1）。

**图1:**
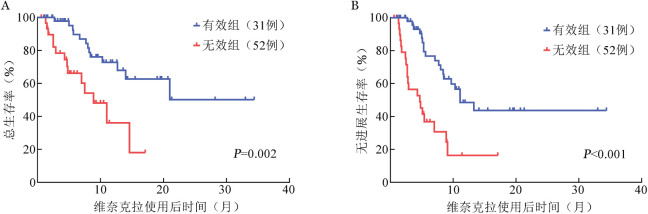
接受维奈克拉＋去甲基化药物治疗的较高危骨髓增生异常综合征患者总生存（A）与无进展生存（B）曲线

## 讨论

较早期MDS的骨髓造血细胞表现出过度凋亡，而较晚期MDS表现为“凋亡阻滞”，凋亡相关调控蛋白，特别是BCL-2在其中发挥重要作用[14]。VEN是一种强效、选择性口服BCL-2抑制剂，通过特异性结合和抑制抗凋亡蛋白BCL-2，使其释放促凋亡蛋白、启动凋亡程序，促使肿瘤细胞凋亡[15]。Jilg等[16]通过体外实验发现VEN可显著诱导HR-MDS患者CD34^+^细胞凋亡，抑制造血干/祖细胞集落形成能力，而对低中危MDS和健康人骨髓CD34^+^细胞没有促凋亡作用。HMA可以使BCL-2水平升高，MCL-1水平降低，有利于VEN的治疗。而且，VEN可能会降低MDS细胞的凋亡阈值，改善其对HMA的治疗反应，甚至在既往HMA耐药的细胞中也是如此[17]。

Ball等[11]的回顾性研究显示，应用VEN+HMA治疗HR-MDS患者，既往未接受HMA、接受过HMA、HMA治疗失败组的ORR分别为75％、62％和44％，获得治疗反应的中位时间为1.6个月；中位随访7.6个月，中位OS期为19.5个月，中位PFS期为15.4个月。VEN+AZA治疗复发/难治 HR-MDS的Ⅰb期临床研究中，中位随访21.2个月，获得CR、mCR、红细胞或血小板脱离输注的比例分别为7％、32％和36％，达到CR/mCR的中位时间为1.2个月，CR/mCR的中位反应持续时间为8.6个月，中位OS期为12.6个月[18]。VEN+AZA作为一线方案治疗HR-MDS Ⅰb期研究的更新结果显示，入组107例患者，中位随访31.9个月，ORR达80.4％，其中CR率为29.9％，mCR率为50.5％，中位OS期为26.0个月，中位CR持续时间16.6个月[19]。本研究中，初治MDS的ORR为66.7％，略低于上述文献报道结果，可能与部分患者随访时间太短，仅观察了1个疗程的疗效有关；HMA治疗失败MDS患者的ORR为53.3％，略高于文献报道结果，可能因为本研究中这部分患者仅15例，病例数较少导致偏倚。

我们试图从常规血液、生化及遗传学指标中找到影响疗效的因素。既往有研究报道，MDS患者骨髓中ALP水平明显增高，并推测其可能与骨髓造血细胞增殖导致的局部骨重塑有关[20]，临床研究也显示在AML和高危MDS患者中，较低的血清ALP水平预示接受化疗后获得 CR的概率更高[21]。本研究也发现ALP<90 U/L的患者，接受VEN+HMA治疗后更容易获得治疗反应。Ball等[11]根据IPSS-R染色体核型分组，发现细胞遗传学极差的患者对治疗的反应较差。我们进行相应分组后，得出两组患者的反应率相似，可能是由于本研究中细胞遗传学极差的患者仅占12.0％，明显低于Ball等的研究。目前研究认为U2AF1突变与MDS患者较差的OS和PFS相关，甚至可能是新发MDS患者的1个独立的预后不良因素[22]。本研究中U2AF1的突变频率与国内报道相近[23]，携带U2AF1突变的患者对VEN+HMA治疗反应较差。目前已知在U2AF1突变的MDS中，包括线粒体损伤、氧化磷酸化、血红素生物合成等在内的多条通路及下游基因表达失调[22]，其是否影响了VEN的促凋亡作用，仍需进一步研究探讨。对于伴TP53突变的AML和MDS患者，DEC 20 mg·m^−2^·d^−1^×10 d有极高的治疗反应率，尽管反应并不持久[24]。在DEC基础上加入VEN, 并未提高伴TP53突变AML患者的总体反应率（66％）和生存期（OS：5.2个月；PFS：3.4个月）[25]。临床前研究表明，TP53突变通过扰乱线粒体稳态和细胞代谢，包括增加氧化磷酸化，赋予对VEN的内在抗性[26]。本研究中，伴随TP53突变的患者应用VEN+HMA治疗有效率仅33.3％，显著低于无TP53突变的患者，可能是由于本研究中仅14.5％的患者应用VEN+DEC治疗且DEC给药剂量较低。结合先前研究结果，提示目前VEN+HMA的两药方案，可能并不能改善TP53突变MDS的治疗反应和结局，伴TP53突变的MDS仍然是一个挑战，临床迫切需要新的治疗方法。

本研究中VEN+HMA治疗最常见的AE为血液学AE和肺部感染，其次为消化道反应和全身症状，与既往报道一致。少数患者在治疗过程中出现严重而漫长的骨髓抑制，应根据患者的骨髓缓解及血象恢复情况，在第2个疗程适当缩短用药天数。尽管VEN+HMA治疗带来高反应率，作为桥接治疗，使更多患者获得allo-HSCT的机会，但本研究中获得治疗反应的患者中仅10例（19.2％）接受allo-HSCT，这可能与患者整体年龄高、合并症多等多种因素有关。VEN+AZA治疗反应持续时间为8.6～16.6个月[18]–[19]，说明多数患者在启动治疗后1年左右复发，所以克服耐药、发掘多药联合及新药治疗方案，仍是今后探索的方向。

本研究有以下局限性：为单中心回顾性研究；未能将初治MDS和难治/复发患者单独分析；治疗方案不严格统一；未能根据VEN血浆药物浓度调整给药剂量；随访时间相对较短，生存期数据不足。相关结果仍需要多中心前瞻性研究来证实。

综上，VEN+HMA治疗HR-MDS有较好的疗效和安全性，对初治MDS治疗反应率较高，对既往HMA治疗失败的患者依然具有较高的治疗反应率。ALP≥90 U/L、伴TP53突变或U2AF1突变的患者可能治疗反应较差。
